# Anthropogenic plutonium-244 in the environment: Insights into plutonium’s longest-lived isotope

**DOI:** 10.1038/srep21512

**Published:** 2016-02-22

**Authors:** Christopher R. Armstrong, Heather A. Brant, Patterson R. Nuessle, Gregory Hall, James R. Cadieux

**Affiliations:** 1 Nonproliferation Technology Section, Savannah River National Laboratory, Aiken, SC, 29808, USA

## Abstract

Owing to the rich history of heavy element production in the unique high flux reactors that operated at the Savannah River Site, USA (SRS) decades ago, trace quantities of plutonium with highly unique isotopic characteristics still persist today in the SRS terrestrial environment. Development of an effective sampling, processing, and analysis strategy enables detailed monitoring of the SRS environment, revealing plutonium isotopic compositions, e.g., ^244^Pu, that reflect the unique legacy of plutonium production at SRS. This work describes the first long-term investigation of anthropogenic ^244^Pu occurrence in the environment. Environmental samples, consisting of collected foot borne debris, were taken at SRS over an eleven year period, from 2003 to 2014. Separation and purification of trace plutonium was carried out followed by three stage thermal ionization mass spectrometry (3STIMS) measurements for plutonium isotopic content and isotopic ratios. Significant ^244^Pu was measured in all of the years sampled with the highest amount observed in 2003. The ^244^Pu content, in femtograms (fg = 10^−15^ g) per gram, ranged from 0.31 fg/g to 44 fg/g in years 2006 and 2003 respectively. In all years, the ^244^Pu/^239^Pu atom ratios were significantly higher than global fallout, ranging from 0.003 to 0.698 in years 2014 and 2003 respectively.

With a half-life of 81 million years, plutonium-244, the longest-lived plutonium (Pu) isotope, can be produced both naturally and by anthropogenic means. Among the plutonium isotopes, ^244^Pu carries the unique distinction of being both relatively heavy and stable (by contrast the half-lives of its heavier counterparts ^245^Pu, ^246^Pu, and ^247^Pu are 10.5 hours, 10.9 days, and 2.3 days respectively). These unique properties, together with advanced analytical techniques, make it possible to monitor and delineate natural and anthropogenic ^244^Pu in the terrestrial environment.

The genesis of ^244^Pu in nature occurs as the result of r-process nucleosynthesis, e.g., in supernova(s) and neutron star mergers^1^,^2^,^3^, some of which contributed to the formation of our Solar System over 4.6 billion years ago^4^. Although the ^244^Pu once present in the Earth’s crust has decayed to undetectable levels^5^,^6^, and the neutron fluxes are insufficient for ^244^Pu to be produced naturally in uranium ores, this isotope has been observed as a galactic hitchhiker on Earth in very small quantities in the relatively pristine deep-sea environment, e.g., in sediments^7^,^8^ and manganese and iron-manganese encrustations^3^,^9^. This rare nuclide has also been found in meteorites^10^,^11^ and in lunar rock samples^12^,^13^.

Anthropogenic ^244^Pu owes its presence in the biosphere to high flux reactors and thermonuclear detonation events respectively. Although anthropogenic ^244^Pu occurrence in the environment is expected to greatly exceed that of its natural counterpart, it is typically found in very small amounts; and studies of ^244^Pu in the environment are scarce. Indeed the first (and only) atmospheric global fallout ratio for ^244^Pu (^244^Pu/^239^Pu = 5.7 × 10^−5^) has only recently been put forth^14^. In addition to its scarcity, analysis of ^244^Pu by conventional means, i.e., alpha spectrometry and mass spectrometry, is a challenge. For context, the radiological properties of plutonium isotopes 236 through 244 are shown in Table 1. Owing to its relatively small specific activity (0.7 GBq/kg), ^244^Pu measurement by alpha spectrometry is generally impractical. Additionally, ^244^Pu is commonly used as an internal tracer for plutonium analysis by mass spectrometry; and in such cases ^244^Pu cannot be measured accurately.

The main reason for the dearth of anthropogenic ^244^Pu in the environment is because it requires very unlikely conditions to be produced: since neutron capture by ^242^Pu produces ^243^Pu, timely neutron capture must also occur by ^243^Pu before it beta decays, with a roughly 5 hour half-life, to americium-243. Man-made production of ^244^Pu therefore can only occur under extreme conditions, and typically via one of two pathways: In a high flux reactor (≥5 × 10^15^ neutrons per square centimeter per second) with a heavy isotope target - conditions not typically encountered in commercial or weapons production reactors - or during a thermonuclear weapon detonation.

## The need for plutonium-244

The world’s stock of ^244^Pu is rapidly depleting. This is a concern primarily because, as mentioned previously, ^244^Pu is a common internal standard used in isotope dilution mass spectrometry for plutonium analyses. It is also an invaluable target material in the production of superheavy elements^15^. Moreover, because of its long half-life it poses significantly less radiation hazard than other plutonium isotopes; therefore, ^244^Pu is an attractive isotope for basic research studies of plutonium. The current shortage and prohibitive cost of making more material (billions of US dollars in over a 50 year timescale^16^), has prompted the only U.S. supplier of plutonium certified reference materials (CRM), New Brunswick Laboratories (NBL), to stop selling its ^244^Pu CRM^17^.

To meet the growing need for this precious commodity, recent interest has been sparked to harvest the isotope from legacy materials, for example, from targets that were irradiated in the high flux reactors at the Savannah River Site USA (SRS) during the heavy isotope production campaigns decades ago. Examples of these materials include the Mark-42 and Mark-18 targets. The Mark-42 targets, containing ^239^Pu as the seed material, were designed to produce ^242^Pu, americium-243 (^243^Am), and curium-244 (^244^Cm). These nuclides were often recycled and incorporated into subsequent target materials to undergo further irradiations to produce californium-252 (^252^Cf). Californium-252 is an ideal neutron source for a variety of medical and industrial applications. The Mark-18 targets contained ^242^Pu. These targets were designed for the sole purpose of producing californium-252 (^252^Cf). At present, the most promising candidates for ^244^Pu recovery are the Mark-18 (Mk-18 A) targets. From August 1969 until November 1970, eighty-six Mk-18 A targets were irradiated in a high-neutron-flux mode in the 2000 megawatt-thermal (MWt) K-Reactor at SRS^18^,^19^. This intense exposure resulted in irradiated Mk-18 A targets with very unique isotopic contents. Upon removal from the reactor, 21 targets were processed at Oak Ridge National Laboratory (ORNL) in 1972–1973 to recover ^252^Cf, heavy curium (^246^Cm through ^248^Cm), and plutonium. The plutonium fraction, rich in ^244^Pu, was electromagnetically separated in the calutrons at ORNL to produce gram quantities of 98–99% ^244^Pu. NBL obtained some of this material to produce the aforementioned ^244^Pu CRM. Other high-purity samples of ^244^Pu were made available to scientists for basic research and for safeguards programs^19^. The remaining Mk-18 A targets, expected to contain 21 grams of ^244^Pu - greater than 90% of the world’s inventory - are currently being stored at SRS. Recognizing the importance of this unique material, in 2001, the United States Department of Energy (DOE) designated the ^244^Pu in the remaining targets as a national resource material^20^. Fueled by demand, researchers at Savannah River National Laboratory (SRNL) and ORNL have recently proposed a plan, currently under development, to process these remaining targets to recover ^244^Pu and heavy curium^17^,^19^.

## Historical studies of plutonium-244

**Figure 1:**
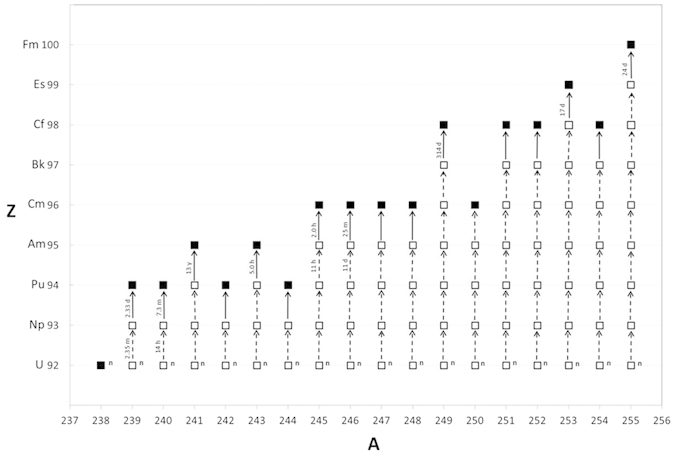
Production of uranium isotopes in the November 1952 Mike thermonuclear event and the associated beta decay products. Modified from Diamond *et al.* 1960^21^. ▫ beta unstable nuclide. ■ beta stable nuclide.

At present the only detailed studies of anthropogenic ^244^Pu in the environment are related to the thermonuclear weapons testing in the northern Marshall Islands^14^,^21^,^22^,^23^,^24^. Between 1946 and 1958, the United States conducted 43 thermonuclear detonation tests at Enewetak and Bikini atolls. These tiny land masses continue to provide some of the few locations where measurable quantities of heavy elements (and isotopes) can be sampled. For example, an examination of a sample related to the 10.4 megaton Ivy Mike test^25^ detonated on November 1, 1952, lead to the discovery of ^244^Pu (einsteinium (Es) and fermium (Fm) were also discovered^22^). A follow up study^21^ examined debris from the Mike test fallout and provided equally fruitful results. Diamond *et al.* noted that the Mike explosion produced, via neutron capture by ^238^U, uranium isotopes ^239^U to ^255^U (inclusively). Measurements of the beta-decay products of these short half-life isotopes resulted in the identification of a plethora of heavy elements and associated isotopes produced from the Mike test (Fig. 1), including a ^244^Pu/^239^Pu atom ratio of (1.18 ± 0.07) × 10^−3^.

More recent anthropogenic ^244^Pu investigations of this sample area have provided additional ^244^Pu/^239^Pu results. A 2010 study^24^ of soils collected from the Bikini atoll presented ^244^Pu/^239^Pu atom ratios ranging from roughly 2–5 × 10^−4^. In another study^23^, a soil sample collected from Enewetak atoll from the Fig-Quince tests exhibited a ^244^Pu/^239^Pu atom ratio of 3.2 × 10^−5^. A contributing factor to the relatively small ratio observed in this latter study is the low fission yields generated in some of these tests^23^. In these cases relatively low ^244^Pu production is expected.

## Plutonium-244 production at the Savannah River Site (SRS)

**Figure 2:**
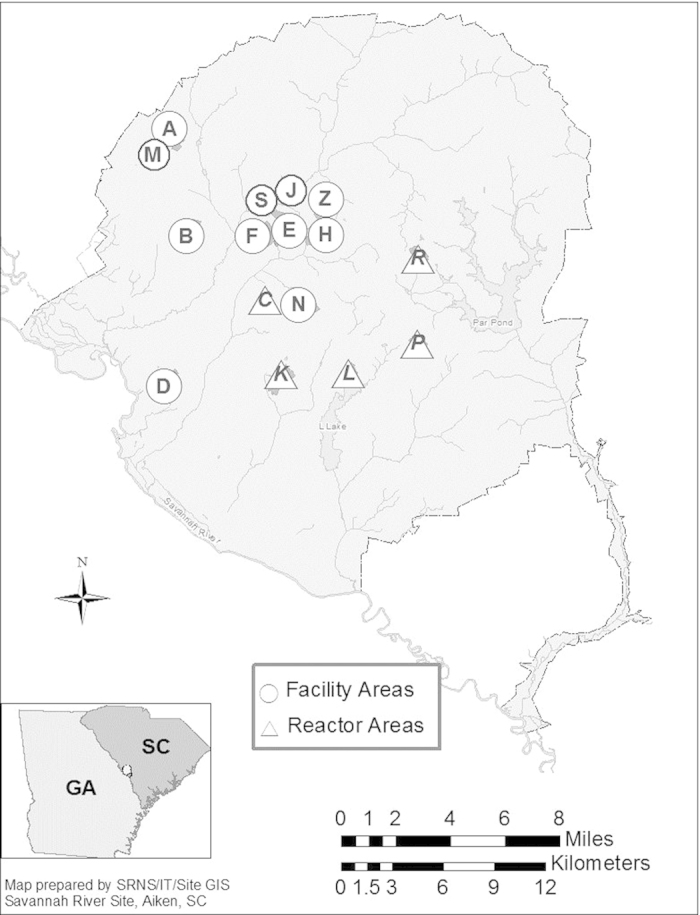
Map of Savannah River Site (SRS) located in South Carolina, USA. Triangles show previous reactor locations (C, K, L, P, and R reactors). Circles show other locations of interest including A: A-Area comprising the Savannah River National Laboratory (SRNL); M: Fuel Fabrication Facility (decommissioned); H: H-Canyon and Tritium Reprocessing Facilities; K: Plutonium storage facility (formerly K reactor).

The Savannah River Site (SRS), formerly the Savannah River Plant (SRP) began operation in the early 1950’s. The site, located close to the Savannah River in South Carolina (USA), encompasses roughly 800 square kilometers (Fig. 2). Its primary purpose was to produce special nuclear materials for national defense, specifically weapons-grade Pu and tritium (^3^H) required for thermonuclear devices^26^. Over its history the site produced metric tons of plutonium comprising Pu isotopes with masses 238 through 244. Former and current plutonium and tritium production and processing facilities are shown in Fig. 2 including Savannah River National Laboratory (SRNL). For over 60 years SRNL, located in A-area at SRS, has performed a variety of research and development functions to support operations at SRS facilities including F-Canyon (decommissioned) and H-Canyon used nuclear fuel reprocessing facilities.

The highly versatile reactors at SRS provided a unique breeding ground for ^244^Pu, as this isotope was produced in the high flux reactors as a byproduct of the californium-252 production campaigns. Notably, during the aforementioned 1969–1970 period, the most ^252^Cf ever made, some 2.1 g, was produced in the SRS K-Reactor^27^ (Fig. 2). To accomplish this feat, targets with ^242^Pu as the source nuclide (including the aforementioned Mark-18 targets) were introduced into the high flux reactor. Taking advantage of the unique capabilities inherent to the SRS reactors, many other transplutonium isotopes were also produced at SRS including curium-244 (which was initially proposed as a deep-space heat source and later abandoned in favor of plutonium-238), and its heavier isotopes, e.g., curium-246–248^17^ as well as isotopes of berkelium (Bk), einsteinium (Es), and fermium (Fm)^28^.

## Aim

Owing to its remarkable plutonium production history^26^, SRS offers a unique environment for detailed study of plutonium fate and transport. We recently reported findings of a long-term study of plutonium detailing sampling, radiochemical processing, and analysis of Pu (^238^Pu through ^242^Pu) in environmental samples from SRS^29^. In this present contribution, we focus specifically on ^244^Pu. This current contribution expands on this work, and here we report ^244^Pu results from sample collections spanning eleven years. In contrast to the aforementioned studies of anthropogenic ^244^Pu related to thermonuclear weapon testing^21^,^23^,^24^,^25^, this current study reports the occurrence of reactor-produced ^244^Pu in the terrestrial environment. Collectively, these studies afford unique insights into the fate and transport of this rare isotope.

## Results

### Plutonium isotopic content

**Figure 3:**
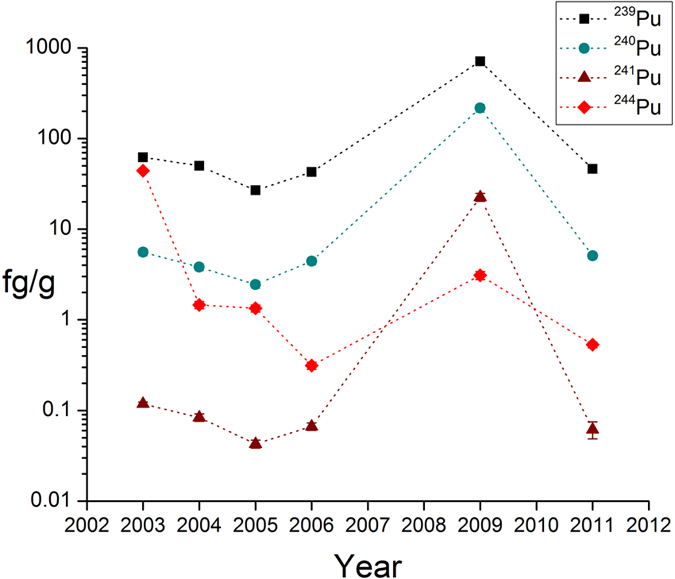
Plutonium isotopic content in shoe brush debris samples from three stage thermal ionization mass spectrometry (3STIMS) measurements (logarithmic scale) in femtograms per gram with two-sigma error. Data are reported for sample years in which a ^242^Pu internal standard was added to the samples.

Samples consisting of foot borne debris removed via a mechanical shoe brush were collected and processed from 2003 through 2014. For years in which a ^242^Pu internal standard (tracer) was added during radiochemical processing (2003, 2004, 2005, 2006, 2009, and 2011), the plutonium isotopic content was quantified for isotopes ^239^Pu, ^240^Pu, ^241^Pu, and ^244^Pu by three stage thermal ionization mass spectrometry (3STIMS). The isotopic mass data for these samples, in femtograms (fg = 10^−15^ g) per gram, are shown in Fig. 3 on a logarithmic scale, and reported in Table 2. Although measurable quantities of all isotopes investigated were observed in all years, mass results of isotopes ^239^Pu, ^240^Pu, and ^241^Pu are included in Fig. 3 and Table 2 for comparative purposes only (see Discussion). Significant amounts of ^244^Pu were observed in all years, with amounts ranging from 0.31 to 44.23 fg/g in years 2006 and 2003 respectively.

### Plutonium atom ratios

Atom ratio data for ^240^Pu/^239^Pu, ^241^Pu/^239^Pu, ^242^Pu/^239^Pu, and ^244^Pu/^239^Pu for this eleven year study are reported in Table 3. The ^240^Pu/^239^Pu, ^241^Pu/^239^Pu, and ^242^Pu/^239^Pu isotopic ratios reported up to 2013, depicted as the shaded region in Table 3 and included for comparison, have been reported previously in our initial report^29^. With the exception of the 2009 sample, in which a pronounced higher burnup signature is observed, collectively the ^240^Pu/^239^Pu isotopic data generally appear to be representative of a variable admixture of fallout and weapons-grade plutonium from local operations, with several ratios approaching weapons-grade Pu (<7% ^240^Pu). Although the ^241^Pu/^239^Pu data also feature an exceptionally high ratio in 2009, all other measured ratios are roughly similar to atmospheric global fallout, reflecting the decay of this relatively short half-life isotope (Table 1) from decades-old SRS material. The ^242^Pu/^239^Pu ratios (for years in which ^242^Pu was measured) are uniformly above fallout, consistent with an admixture of fallout and higher burnup material. Elevated ^244^Pu/^239^Pu atom ratios were also measured in every sample year relative to atmospheric fallout (Table 3), but to a larger extent than observed in the ^242^Pu/^239^Pu data. Similarly to the data reported in Table 2, the 2003 sample year featured the most pronounced ^244^Pu enrichment, exhibiting a ^244^Pu/^239^Pu atom ratio of approximately 0.7.

## Discussion

### Plutonium-244 in the Savannah River Site (SRS) environment

Significant and highly variable ^244^Pu was observed throughout the eleven year period of this study. Due to the sheer volume of foot traffic that passes through the shoe brushes (hundreds of people annually from various A-Area locations and SRS in general), and the range of materials collected (soil, rock/mineral fragments, organic material, etc.) variability in plutonium-244 collections from year to year is expected. However, despite this variability, every collection year significantly exceeded the atmospheric fallout value.

The presence of significant ^244^Pu in all sample years can be attributed to the rich history of transuranic isotope production at SRS. The proximity of associated radiochemical processing activities to our clean laboratories in which the shoe brush sample collectors are housed may also be a key factor. As mentioned previously, ^244^Pu was produced in the SRS high flux reactors decades ago. The irradiated targets containing ^244^Pu, ^244^Cm, ^252^Cf, etc., were stored and/or processed in F-Area and at the Savannah River Laboratory (SRL)^28^, now SRNL, in A-Area (Fig. 2). This latter location is located a short distance (<½ km) from our A-Area clean laboratories. This long-term dataset demonstrates a unique ^244^Pu signature, reflective of this production history, which continues to persist in the SRS local environment today.

The most striking collection years occurred in 2003, in which the most elevated ^244^Pu was observed, and in 2009, which featured anomalously high ^239^Pu, ^240^Pu, and ^241^Pu. The high ^244^Pu observed in the 2003 sample is likely due to activities associated with high activity waste (HAW) processing in the SRNL A-Area facility, located less a half kilometer from our cleanroom facilities (where the shoe brush sample collectors are located). In the early 2000’s a decision was made by DOE to initiate disposition of HAW stored in F-Area (Fig. 2) at SRS. This HAW contained appreciable quantities of transuranic isotopes, particularly americium and curium, associated with the targets that had been irradiated in the high flux SRS reactors, e.g., K-Reactor, decades earlier. This material had been in temporary storage for decades in F-Area. Disposal involved converting the material into a borosilicate glass, i.e., vitrification, at the SRS Defense Waste Processing Facility (DWPF) for permanent storage. Due to the high activity of this material^30^, relatively small batches were sent to the Shielded Cells Facility in A-Area to undergo characterization studies prior to vitrification at DWPF. In 2002, a detailed analysis (including alpha spectrometry) was conducted on this material and significant quantities of ^243^Am and ^244^Cm were measured (unpublished data). Although ^244^Pu was not analyzed, a significant quantity of this isotope is also expected to be present in that 2002 HAW sample. Thus, the timing of this HAW processing campaign is consistent with the 2003 sample collection that yielded the high ^244^Pu. The marked elevation in ^240^Pu and ^241^Pu relative to ^239^Pu in 2009 was most likely due to plutonium oxide processing activities that occurred during this time frame in an adjacent laboratory in A-Area at SRNL^29^,^31^. These materials contained relatively high ^240^Pu and ^241^Pu; and it is conceivable that, similarly to the high ^244^Pu collection in 2003, a small contribution was tracked into our laboratory wing entrance and picked up by the shoe brushes. Thus an examination of this complete dataset reported in Table 3 provides clear evidence of both the weapons-grade Pu and the heavy element production legacies at SRS. Further, the persistence of these materials offers unique insights into plutonium fate and transport in the local SRS environment.

### Comparison to other studies

Little data from reactor-produced ^244^Pu in environmental samples are currently available. As such, comparison to anthropogenic ^244^Pu data is limited to either samples associated with thermonuclear weapon tests or potentially reactor-produced ^244^Pu that is difficult to distinguish from global fallout. The ^244^Pu/^239^Pu atom ratios reported in Table 3 are significantly higher than the values reported from the most recent Marshall Island studies^32^,^33^. Moreover, the lowest ^244^Pu/^239^Pu ratio observed in this study (~0.003 in 2014) is greater than the ^244^Pu/^239^Pu atom ratio observed in the earlier collections following the Mike thermonuclear detonation test ((1.18 ± 0.07) × 10−3 (21)). Recent environmental samples obtained from the Sellafield nuclear reprocessing facility in the United Kingdom feature ^244^Pu/^239^Pu atom ratios^14^ consistent with global fallout. The quantities of ^244^Pu measured in our study are representative of a sample set that is unique to the SRS environment, where gram quantities of ^244^Pu were made in the specially-designed high flux reactors that once operated at SRS. Although production and processing of ^244^Pu ceased decades ago, re-suspension of these legacy materials resulted in a general dispersion of ^244^Pu containing solid phases such that small quantities are still readily quantifiable.

## Methods

### Methodology

The environmental samples in this study consist of materials collected from the bottom of visitors’ footwear via a mechanical shoe brush^34^ before entering the cleanroom facilities (Class 1000–10,000) at Savannah River National Laboratory (SRNL). The shoe brush (Liberty Shoe Brush 2010SC; Liberty Industries Inc., East Berlin, CT, USA) mechanically removes foot borne debris via four rotating brushes that contact the front, sides, and bottom of the footwear in conjunction with a vacuum system. Further sample collection details are provided in a previous study^29^. Briefly, collections consist of a variety of common terrestrial materials, including aerosol particles, mineral fragments, vegetation debris, soil, etc. For all intents and purposes, no size or shape discrimination occurs with this collection method. Observed sizes of collected materials range from tens of microns to single centimeters. This sampling method offers a unique opportunity to collect bulk samples that are representative of the surrounding SRNL (A-Area; Fig. 2) environment. Samples are collected periodically (typically once or twice per year) and processed via the SRNL radiochemical processing procedure specifically designed for trace level plutonium purification and separation^29^. The separated and purified plutonium samples are analyzed first by alpha spectrometry and then by mass spectrometry for total plutonium and plutonium isotopic composition determination.

### Sample preparation and mass spectrometry

All glass, quartz, and Teflon materials are pretreated (leached) by refluxing for several hours in 8 M nitric acid. Type 1 (18 MΩ) water and high purity semiconductor grade acids are used exclusively in this study. All work was conducted in Class 10,000 and Class 1,000 clean rooms.

Experimental details related to this study have been described previously^29^. This plutonium separation and purification approach is specifically tailored to eliminate impurities and potential mass spectrometry isobaric interferences, e.g., from curium-244. Briefly, upon extraction from the shoe brushes, samples are mixed manually and approximately 100 grams are subsampled and weighed out. All samples are ashed for at least eight hours in a furnace at 600 °C to destroy organic components in the matrix. The samples are subsequently digested and centrifuged in accordance with a well-established method^32^, and the solid residue is archived for periodic monitoring of refractory Pu. The sample is transferred by weight to a leached pyrex beaker and spiked with ^242^Pu (15–20 pg from NIST SRM 4334 G). The remaining solution is archived. After an oxidation state adjustment (to Pu(IV)) and a co-precipitation step trace plutonium purification is performed according to an original methodology^33^ whereby samples are loaded and eluted through successive columns of AG1 X 8 resin (Eichrom) and AG1 X 4 (Eichrom) ion exchange resins.

### Three stage thermal ionization mass spectrometry (3STIMS)

Plutonium measurements were conducted with a 1960 s-era KAPL (Knolls Atomic Power Laboratory) design Three Stage Thermal Ionization Mass Spectrometer (3STIMS) fabricated in-house in the 1970’s. The instrument uses three 90° × 30.5 cm sectors arranged in magnetic/magnetic/electrostatic order (momentum/momentum/energy filter order) with a single ion counting detector. This arrangement allows for measurement of very high ratios between adjacent ion masses, of order 10E8, but the instrument must be sequentially tuned from mass to mass to collect ions at each isotope, less efficient than an instrument with a detector per ion beam. It routinely analyzes nanogram mass U samples and picogram mass Pu samples. Purified samples are loaded onto anion exchange resin beads, which are then loaded by hand onto high-purity rhenium filaments and placed in the source region of the mass spectrometer for thermal ionization.

### 3STIMS measurements

Isotopic content measurements of ^239^Pu, ^240^Pu, ^241^Pu, and ^244^Pu together with atom ratios of ^240^Pu/^239^Pu, ^241^Pu/^239^Pu, ^242^Pu/^239^Pu, and ^244^Pu/^239^Pu were carried out by three-stage thermal ionization mass spectrometry (3STIMS). These data are corrected for small impurities (trace ^238–241^Pu) in the NIST SRM 4334 G standard. All data are mass bias corrected, thus reducing systematic error. Stringent quality assurance and quality control measures are routinely undertaken with the 3STIMS instrument. A two-sigma relative standard deviation (RSD) of approximately 10% is expected for measurements of Pu in the 10 fg (fg = 10^−15^ g) range. In addition to employing internal standards, an external standard (certified ratio standard: New Brunswick CRM-128) consisting of 10–30 pg total Pu of a 1:1 ^239^Pu:^242^Pu with trace ^238^Pu (<1 fg), ^240^Pu and ^241^Pu (on the order of single fg) is ran with every sample set. For Pu isotopic abundances excellent accuracy is observed with this instrument. For example, when compared to the certified (CRM-128) value, the 239/242, 240/242, and 241/242 ratios are routinely within analytical error (typically 0.5% or better).

### Quality assurance and quality control (QA/QC)

Stringent quality assurance and quality control (QA/QC) protocols for trace plutonium analysis in environmental samples have been in place for decades in this laboratory. Reagent blanks (run through complete chemistry with the same amount of SRM 4334 G standard used in actual samples) and bench standards (run through chemistry with ~3 pg of ^240^Pu from NIST SRM 4338 A) were processed with every sample set. The background Pu levels in the laboratory are monitored routinely (via swipe sampling, radiochemical processing and 3STIMS analysis) and are consistently below the analytical detection limit of the 3STIMS. Although the reagent blanks were similarly below the instrument detection limit. In addition, as part of a National Institute of Standards and Technology (NIST) international blind round robin exercise conducted in 2005–2006, we investigated a new NIST standard reference material, Rocky Flats Soil II (SRM 4353 A). The results of this exercise provided data that contributed to the certification of this material^35^. This material represents weapons-grade Pu that was not expected to contain ^244^Pu. Thus this material enables us to establish the low end of detection (~0.006 atom percent ^244^Pu) for a similar sample matrix to the one investigated in this study. A set of replicate samples were spiked with ^242^Pu and a second set was run unspiked, i.e., there was enough ^239^Pu in the samples on which to tune the TIMS instrument. Thus, in addition to routine screening to verify background levels, this exercise enabled us to further verify that the laboratory background contained negligible ^244^Pu. See the Supplementary Information section (Table S.1) for further details.

## Additional Information

**How to cite this article**: Armstrong, C. R. *et al.* Anthropogenic plutonium-244 in the environment: Insights into plutonium’s longest-lived isotope. *Sci. Rep.* **6**, 21512; doi: 10.1038/srep21512 (2016).

## Supplementary Material

Supplementary Information

## Figures and Tables

**Table 1:** Summary of the radiological properties of relevant plutonium isotopes^36^,^37^.

	Pu-236	Pu-238	Pu-239	Pu-240	Pu-241	Pu-242	Pu-244
Half-life (in years)	2.9	87.74	24,110	6537	14.4	375,000	8.1E7
Specific activity (GBq/kg)	2.0E + 7	6.4E + 53	2.3E + 3	8.5E + 3	3.9E + 6	1.5E + 2	7.0E-1
Principal decay mode	alpha	alpha	alpha	alpha (some spontaneous fission)	beta	alpha	alpha
Decay energy (MeV)	5.768	5.593	5.244	5.255	0.021	4.983	4.6
Radiological hazards	alpha, weak gamma	alpha, weak gamma	alpha, weak gamma	alpha, weak gamma	beta, weak gamma	alpha, weak gamma	alpha, weak gamma

**Table 2:** Plutonium isotopic content from three stage thermal ionization mass spectrometry (3STIMS) measurements in femtograms per gram (fg/g) with two-sigma error.

Year	^239^Pu	^240^Pu	^241^Pu	^244^Pu
2003	62.06 ± 5.61	5.57 ± 0.50	0.12 ± 0.01	44.23 ± 2.00
2004	50.09 ± 4.52	3.81 ± 0.34	0.08 ± 0.01	1.46 ± 0.13
2005	26.84 ± 2.42	2.45 ± 0.22	0.04 ± 0.01	1.34 ± 0.12
2006	42.62 ± 3.85	4.45 ± 0.40	0.07 ± 0.01	0.31 ± 0.03
2009	712.88 ± 71.90	217.24 ± 21.91	22.51 ± 2.27	3.10 ± 0.31
2011	46.34 ± 3.43	5.10 ± 0.39	0.06 ± 0.01	0.53 ± 0.04

Data are reported for sample years in which a ^242^Pu internal standard was added to the samples.

**Table 3:**
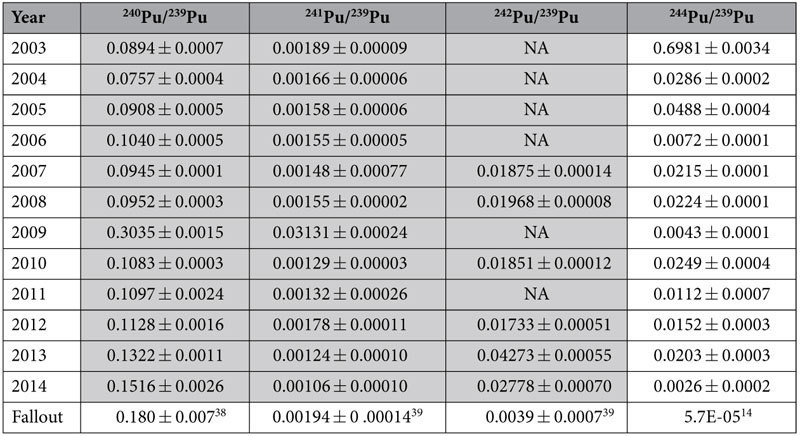
Three stage thermal ionization mass spectrometry (3STIMS) plutonium atom ratios for SRS environmental samples spanning an eleven year period with two-sigma error.

Shaded values were initially reported in Armstrong *et al.* 2015^29^. NA: not applicable (^242^Pu could not be quantified).
