# Supplementary material for: Anthropogenic plutonium-244 in the environment: Insights into plutonium’s longest-lived isotope
Source: Sci Rep. 2016 Feb 22;6:21512. doi: 10.1038/srep21512 (PMC4761908; doi:10.1038/srep21512)
Supplement: Supplementary Information [file srep21512-s1.pdf]

Anthropogenic plutonium-244 in the environment: Insights into plutonium's longest-lived isotope

Christopher R. Armstrong*, Heather A. Brant, Patterson R. Nuessle, Gregory Hall, and James R. Cadieux

Nonproliferation Technology Section, Savannah River National Laboratory, Aiken, SC, USA, 29808

*Corresponding author: christopher.armstrong@srnl.doe.gov

Supplementary Information

Table S.1. Three stage thermal ionization mass spectrometry (3STIMS) plutonium measurements for a series of replicate spiked (15 pg Pu-242) and unspiked Rocky Flats Soil II (SRM 4359A) samples (10 g). Data are represented in atom percentages with 2-sigma error.

	239	2 sigma	240	2 sigma	241	2 sigma	242	2 sigma	244	2 sigma	240/239	2 sigma
Spiked -1	3.514at%	0.026at%	0.196at%	0.003at%	0.005at%	0.001at%	96.280at%	0.455at%	0.005at%	0.001at%	5.59%	0.11%
Spiked -2	5.684at%	0.022at%	0.337at%	0.002at%	0.004at%	0.000at%	93.974at%	0.218at%	0.000at%	0.000at%	5.93%	0.05%
Spiked -3	3.680at%	0.016at%	0.206at%	0.002at%	0.002at%	0.000at%	96.112at%	0.222at%	0.000at%	0.000at%	5.59%	0.05%
Spiked -4	4.709at%	0.021at%	0.268at%	0.002at%	0.003at%	0.000at%	95.019at%	0.258at%	0.000at%	0.000at%	5.70%	0.06%
Spiked -5	3.558at%	0.016at%	0.195at%	0.002at%	0.002at%	0.000at%	96.245at%	0.265at%	0.000at%	0.000at%	5.47%	0.06%
Spiked -6	3.130at%	0.011at%	0.173at%	0.001at%	0.002at%	0.000at%	96.695at%	0.170at%	0.000at%	0.000at%	5.54%	0.04%
Spiked -7	3.497at%	0.012at%	0.195at%	0.001at%	0.002at%	0.000at%	96.306at%	0.179at%	0.000at%	0.000at%	5.56%	0.04%
Spiked -8	3.918at%	0.013at%	0.215at%	0.001at%	0.002at%	0.000at%	95.865at%	0.179at%	0.000at%	0.000at%	5.49%	0.04%
Spiked -9	3.328at%	0.013at%	0.188at%	0.002at%	0.002at%	0.000at%	96.481at%	0.210at%	0.000at%	0.000at%	5.66%	0.05%
Spiked -10	3.292at%	0.011at%	0.187at%	0.001at%	0.002at%	0.000at%	96.520at%	0.164at%	0.000at%	0.000at%	5.67%	0.04%
Spiked -11	2.973at%	0.009at%	0.167at%	0.001at%	0.002at%	0.000at%	96.858at%	0.131at%	0.000at%	0.000at%	5.62%	0.04%
Spiked -12	3.757at%	0.014at%	0.208at%	0.002at%	0.002at%	0.000at%	96.032at%	0.207at%	0.000at%	0.000at%	5.55%	0.05%
Spiked -13	4.118at%	0.021at%	0.224at%	0.003at%	0.002at%	0.000at%	95.655at%	0.326at%	0.000at%	0.000at%	5.45%	0.07%
Spiked -14	4.364at%	0.038at%	0.227at%	0.005at%	0.002at%	0.000at%	95.407at%	0.595at%	0.000at%	0.000at%	5.20%	0.12%
Spiked -15	3.625at%	0.011at%	0.201at%	0.001at%	0.002at%	0.000at%	96.172at%	0.171at%	0.000at%	0.000at%	5.55%	0.04%
Unspiked -1	94.630at%	1.024at%	5.255at%	0.100at%	0.051at%	0.005at%	0.059at%	0.004at%	0.006at%	0.001at%	5.55%	0.12%
Unspiked -2	94.309at%	0.539at%	5.586at%	0.053at%	0.062at%	0.003at%	0.043at%	0.002at%	0.001at%	0.000at%	5.92%	0.07%
Unspiked -3	94.611at%	0.792at%	5.298at%	0.077at%	0.046at%	0.004at%	0.045at%	0.003at%	0.000at%	0.000at%	5.60%	0.09%
Unspiked -4	94.517at%	0.763at%	5.393at%	0.075at%	0.049at%	0.004at%	0.041at%	0.003at%	0.000at%	0.000at%	5.71%	0.09%
Unspiked -5	94.773at%	0.654at%	5.125at%	0.061at%	0.049at%	0.003at%	0.050at%	0.002at%	0.003at%	0.001at%	5.41%	0.07%
Unspiked -6	94.652at%	0.693at%	5.254at%	0.066at%	0.047at%	0.003at%	0.047at%	0.002at%	0.001at%	0.000at%	5.55%	0.08%
Unspiked -7	94.692at%	0.643at%	5.221at%	0.061at%	0.046at%	0.003at%	0.040at%	0.002at%	0.001at%	0.000at%	5.51%	0.07%
Unspiked -8	94.496at%	0.621at%	5.402at%	0.061at%	0.051at%	0.003at%	0.051at%	0.002at%	0.000at%	0.000at%	5.72%	0.07%
Unspiked -9	94.340at%	0.887at%	5.274at%	0.087at%	0.047at%	0.005at%	0.338at%	0.009at%	0.000at%	0.000at%	5.59%	0.11%
Unspiked -10	94.617at%	0.667at%	5.288at%	0.065at%	0.050at%	0.003at%	0.045at%	0.002at%	0.001at%	0.000at%	5.59%	0.08%
Unspiked -11	94.682at%	0.641at%	5.223at%	0.062at%	0.049at%	0.003at%	0.044at%	0.002at%	0.002at%	0.000at%	5.52%	0.07%
Unspiked -12	94.758at%	0.990at%	5.147at%	0.096at%	0.047at%	0.005at%	0.045at%	0.004at%	0.004at%	0.001at%	5.43%	0.12%
Unspiked -13	95.121at%	1.492at%	4.799at%	0.138at%	0.043at%	0.007at%	0.037at%	0.005at%	0.000at%	0.001at%	5.04%	0.17%
Unspiked -14	94.642at%	0.730at%	5.254at%	0.071at%	0.048at%	0.004at%	0.056at%	0.003at%	0.001at%	0.000at%	5.55%	0.09%
